# Editorial: Role of congestion in heart failure: From bench to clinical practice

**DOI:** 10.3389/fphys.2022.1116902

**Published:** 2022-12-20

**Authors:** Jesus Alvarez-Garcia, Paola Morejon-Barragan, Carlos Santos-Gallego

**Affiliations:** ^1^ Ramón y Cajal University Hospital, Madrid, Spain; ^2^ Clínica Guayaquil, Guayaquil, Guayas, Ecuador; ^3^ AtheroThrombosis Research Unit, Mount Sinai Hospital, New York, NY, United States

**Keywords:** congestion, heart failure, lung ultrasound, REDs, biomarker

Congestion plays a central role in the pathophysiology of heart failure (HF) and remains a clinical challenge to detect, prevent, and treat it effectively. Residual fluid overload at the time of discharge, which is frequently underdiagnosed, is one of the main risk factors for readmission (Lala et al., 2015; Rivas-Lasarte et al., 2020). To date, congestion is evaluated through clinical history, physical examination, determination of plasma natriuretic peptide, and X-Ray. However, emerging tools such as ultrasound imaging, remote dielectric sensing technology (ReDS), and new biomarkers such CA 12.5 offer an earlier and more accurate diagnosis. In addition, some of the new drugs for the treatment of HF, such as sacubitril-valsartan or type 2 sodium-glucose cotransporter inhibitors (SGLT2i), have a diuretic effect and the weight of this peculiarity on prognosis has yet to be elucidated.

The aim of this Research Topic on the *“Role of Congestion in Heart Failure: From Bench to Clinical Practice”* is to summarize knowledge on the precise mechanisms involved in the development of congestion in HF, new techniques to assess fluid overload, and to discuss current and emerging treatment approaches to relieve congestion and, ultimately, impact prognosis. The eight original articles included cover a range of Research Topic, from cutting-edge research findings in animal models to the novel tools and treatments used in daily clinical practice.

Han et al. reinforce the prognostic impact of congestive HF exerts on survival in nearly 70,000 patients from Singapore undergoing surgery. Making use of several statistical models they indistinctly observe that congestive HF is an independent risk factor for 1-year mortality after surgery, underscoring the need for optimizing clinical decision-making, improving preoperative consultation, and promoting clinical communication.

Three original articles in the Research Topic discuss the role of ultrasound imaging techniques, underlining the emerging prognostic role of lung ultrasound in different clinical scenarios. Szabó et al. evaluated the prevalence and prognostic value of sonographic pulmonary congestion in 75 consecutive patients with moderate to severe aortic valve stenosis. A third of these patients presented with a high degree of lung congestion and, after a follow-up of 13 months, a number of B-lines ≥30 on ultrasound exam independently predicted a composite outcome including death, hospitalization for HF, and intensification of loop diuretic therapy. Keeping in mind that aortic valve stenosis is, by far, the most common primary valve lesion requiring intervention in Western countries, lung ultrasound offers a promising tool for optimizing the prognostic stratification and timing of valve replacement in a growing population with aortic stenosis. Finally, Maestro-Benedicto et al. analyze the incremental prognostic value of adding the number of B-lines to 4 contemporary HF risk scores applied to a study population from the LUS-HF trial (Rivas-Lasarte et al., 2019). They observed that adding lung ultrasound data evaluated at discharge improved the predictive value of most of the risk scores. Given the fact that lung ultrasound is a relatively simple, fast, and non-invasive test, they recommend incorporating this tool in the risk stratification armamentarium to make medical decisions based on life expectancy and develop appropriate treatment plans.

Two papers investigated new approaches to managing congestion and diuretic resistance. García-Magallón et al. assessed the frequency of HF and the clinical profile of patients with insufficient diuretic response according to the algorithm provided by the 2021 HF European Guidelines (McDonagh et al., 2021). This scheme, based on diuresis and natriuresis, was able to detect up to 29% of patients with diuretic resistance, who had lower systolic blood pressure, worse glomerular filtration rate, higher plasma aldosterone levels, and required more frequent thiazides and inotropes during admission. This is the first study to show the performance of the algorithm for the early assessment of diuretic response in a cohort of patients with acute HF. In addition, Civera et al. evaluated the effect of venous leg compression on short-term changes on intravascular refill, assessed by quantifying inferior vena cava (IVC) diameter in patients with worsening HF requiring parenteral furosemide. They also considered whether early changes in IVC diameter are related to short-term decongestion. Through an exhaustive protocol in 20 patients with congestive HF without signs of intravascular congestion (IVC ≤21 mm) at baseline treated with subcutaneous furosemide, they found that short-term venous leg compression using elastic bandages enhanced the diuretic response. Conversely, it seems to play no role in those with intravascular congestion.

Finally, two exceptional reviews cover the role of congestion in HF from different but complementary viewpoints. Saura et al. discuss several animal models of congestive HF, their advantages, and the limitations of each procedure with respect to the effectiveness of results in terms of clinical application in humans. Rodríguez-Espinosa et al. review the pathophysiological mechanisms involved in cardiorenal syndrome, new tools of biomarkers, or lung, vascular, and renal ultrasound currently being used to detect subclinical fluid overload, and different strategies for treating congestion from a multidisciplinary approach.

This Research Topic aims to increase interest in continuing to advance the pathophysiology, diagnosis, and treatment of congestion, the main feature of most patients with HF.
